# Advancing equity in cancer care: a pilot explanatory mixed methods study of a racially, ethnically, and linguistically concordant model of patient navigation

**DOI:** 10.1080/29944694.2025.2562057

**Published:** 2025-09-29

**Authors:** Ashlyn Tom, Citlali Gomez-Acosta, Vida Henderson, Jean McDougall, Jason A. Mendoza, Elizabeth Carosso, Eliza Brumer Cohn, Wendy E. Barrington, Liszet Bigelow-Chavez, Yaw A. Nyame, K. Casey Lion

**Affiliations:** a Department of Health Systems and Population Health, University of Washington School of Public Health, Seattle, WA, USA;; b Fred Hutchinson Cancer Center, Seattle, WA, USA;; c Division of Public Health Sciences, Fred Hutchinson Cancer Center, Seattle, WA, USA;; d Office of Community Outreach and Engagement, Fred Hutchinson Cancer Center, Seattle, WA, USA;; e Cancer Prevention Program, Public Health Sciences Division, Fred Hutchinson Cancer Center, Seattle, WA, USA;; f Department of Pediatrics, University of Washington School of Medicine, Seattle, WA, USA;; g Department of Child, Family, and Population Health, University of Washington School of Nursing, Seattle, WA, USA;; h Department of Epidemiology, University of Washington School of Public Health, Seattle, WA, USA;; i Department of Urology, University of Washington School of Medicine, Seattle, WA, USA;; j Center for Child Health, Behavior, and Development, Seattle Children’s Research Institute, Seattle, WA, USA;; k Center for Health Outcomes, Seattle Children’s Hospital, Seattle, WA, USA

**Keywords:** Patient navigation, cancer disparities, racial concordance, linguistic concordance, navigator concordance, health equity

## Abstract

Disparities in cancer outcomes persist among systemically marginalized patients. A new racially, ethnically, or linguistically concordant (RELC) model of patient navigation (PN) was piloted at Fred Hutchinson Cancer Center in 2019. An explanatory mixed-methods observational study assessed patient-centered outcomes of RELC PN, traditional PN, and no PN. Patients from these models completed surveys at baseline and follow-up to measure changes in satisfaction, discrimination, resilience, stress, trust, and discussion of clinical trials. Interviews with patients receiving RELC PN were analyzed using thematic analysis. A total of 118 participants completed surveys. Satisfaction with care improved by 4.2 points (SD 7.0) on an 18-item 5-point Likert scale for those receiving RELC navigation, with no change in traditional or no PN groups. Discrimination based on race dropped from 40% (*n* = 2) to 20% (*n* = 1) in the RELC model. A higher proportion of RELC PN patients (80%; *n* = 4) discussed clinical trials compared to traditional PN (17%; *n* = 3) and no PN (20% *n* = 19). Thematic analysis of 29 interviews indicated the model was crucial in overcoming racism, improving trust, and empowering patients. This study highlights the potential of RELC PN to improve patient satisfaction, increase participation in clinical trials, and reduce experiences of discrimination.

## Background

Despite substantial progress in cancer prevention and treatment over the past few decades, disparities in cancer outcomes persist among patients with low incomes and from minoritized racial and ethnic backgrounds (Kolb et al., 2006; Parikh-Patel et al., 2017). These disparities result from historical processes of racialization and racism within both our society and healthcare system resulting in long-standing and deeply rooted discrimination (Abuali et al., 2023; Goel et al., 2022; Hamed et al., 2022; Zubizarreta et al., 2025). The term minoritized is used to refer to groups that are marginalized due to unequal power dynamics (Hamed et al., 2022; Williams et al., 2024). Racism manifests in delays in care, biased communication and medical decision making, poor care coordination, and limited financial resources all contributing to greater financial toxicity and lower care quality (Abuali et al., 2023; Chen et al., 2023; Islami et al., 2024; Jean-Pierre et al., 2011; Kolb et al., 2006; Merz et al., 2024; Mitchell et al., 2022; Montes de Oca et al., 2023; Morris et al., 2010; Parikh-Patel et al., 2017; Patel et al., 2020; Penner et al., 2016; Roy et al., 2021; Ward et al., 2004; Williams et al., 2024; Zubizarreta et al., 2025). Consequently, low-income and minoritized patients experience worse outcomes across a range of cancer diagnoses, leading to delays and lower quality of care reported for individuals on the basis of insurance type, race and ethnicity (Goel et al., 2022; Islami et al., 2024; Mitchell et al., 2022; Parikh-Patel et al., 2017; Zavala et al., 2021).

In the catchment area for the Fred Hutch/University of Washington/Seattle Children’s Cancer Consortium, a National Cancer Institute (NCI) designated Comprehensive Cancer Center in Washington state, African-American(B/AA) and American Indian/Alaska Native (AI/AN) have a higher cancer incidence and death rates for several cancer types compared to other ethnic groups (Office of Community Outreach & Engagement, 2023). Although Hispanic/Latinos have lower incidence rates, they still face higher death rates from several cancer types. Moreover, mortality from breast cancer, colorectal cancer and hematologic malignancies were highest in both AI/AN and B/AA and prostate cancers had the highest mortality rates in B/AA (Office of Community Outreach & Engagement, 2023). A study in Washington state demonstrated that the lowest income neighborhoods experienced the highest incidence of late-stage cancer diagnosis, disproportionately affecting B/AA and Asian and Pacific Islander (AA/PI) populations (Fred Hutch Cancer Center, 2019). These disparities demonstrate a need for interventions to support equitable outcomes for minoritized patients.

Patient navigation (PN) is one strategy that has been developed to reduce cancer disparities (Freeman & Rodriguez, 2011; Jang et al., 2025; Natale-Pereira et al., 2011). Patient navigators help coordinate treatment and remove barriers to care to improve treatment timeliness, adherence, and satisfaction (Bernardo et al., 2019; Chan et al., 2023; Denver Patient Navigation Research Program, 2012; Jean-Pierre et al., 2011; Nelson et al., 2020; Post et al., 2015; Schwaderer & Itano, 2007; Wells et al., 2018; Writing Group of the Patient Navigation Research Program, 2014). In 2007, the Fred Hutchinson Cancer Center (FHCC) created a PN program to address barriers to care, especially those related to tangible resources. The PN program at FHCC offered episodic assistance prompted by an acute concern and referral. Although the program has been successful, there were concerns that minoritized patient populations often disparately affected by poor cancer outcomes were being missed and that their concerns could be addressed proactively to prevent crises. To address this gap, we developed and piloted a new racially, ethnically, and linguistically concordant (RELC) model of cancer navigation in 2019. This new model offers proactive, longitudinal navigation with a focus on racial and linguistic concordance aiming to extend support, improve trust, and ensure timely follow-up to patients from groups known to experience cancer care disparities. Numerous studies have shown that concordance of providers based on shared racial, ethnic, or linguistic backgrounds, can help reduce the effects of racism by promoting trust and facilitating culturally sensitive care, addressing biases and barriers that frequently contribute to health disparities (Blanchard et al., 2007; Charlot et al., 2015; Guadagnolo et al., 2011; Jandorf et al., 2013; Lion et al., 2024; Moore et al., 2023; Muliira & D’Souza, 2016; Paskett et al., 2016). For example, one recent study found that race concordance was associated with shorter times to diagnosis among patients undergoing breast and cervical cancer treatment (Charlot et al., 2015). Another study examining Black patients’ perceptions of racial concordance found that those who preferred race-concordant providers reported greater feelings of comfort, safety, relatability, and cultural understanding (Moore et al., 2023).

To understand if this new model improved patient satisfaction, experiences of healthcare discrimination, and patient-reported outcomes, we conducted an explanatory mixed method, observational study to characterize the experience of patients receiving the new RELC model, the traditional acute model, and those who did not receive navigation. Additionally, to gain deeper insight of patient perspectives and to better understand our survey results, we conducted patient interviews with those who received the RELC model of PN.

### Culturally, racially, and linguistically concordant patient navigation program

The traditional, acute model of PN offered services to any patient at any point in their cancer care based on a provider referral or positive response to tangible needs questions on one of two screening questionnaires in use during the study: the Universal Screening tool or Supportive Care Cancer Questionnaire (SCCQ). The Universal Screening tool had 43 items available in multiple languages via paper and email while the SCCQ had 38 items available only in English via app or web. Despite the screening and referral process, this approach may have missed patients who could benefit from navigation services as the length of each questionnaire created a barrier for patients and completion rates ranged between 40 and 50%.

The RELC model of PN was developed to foster trust and connection using a longitudinal model of care (Chisholm et al., 2022; Lion et al., 2024) that proactively identified and offered racially, culturally, or linguistically concordant navigator services to patients identifying as AI/AN, B/AA, AA/PI, or Spanish-speaking. These patient populations were chosen given known cancer outcome disparities, a long-standing history of experiencing racism and discrimination within and outside of the healthcare setting, and barriers to care frequently internally reported for these groups (Barocas et al., 2014; Benjamins et al., 2016; Lee et al., 2018; Parikh-Patel et al., 2017; Schmid et al., 2016; White-Means & Osmani, 2017; Yedjou et al., 2017). This updated approach differs from the traditional method by identifying and offering longitudinal support to patients who may not have otherwise received a referral to PN, recognizing that the referral-based model required patients to ask a provider for help (requiring trust and awareness that help might be available) or complete a long written intake questionnaire (requiring literacy, specific language skills, digital access, and the time and ability to prioritize something that might not seem essential to someone grappling with a cancer diagnosis).

Navigators who identified as AI/AN, B/AA, AA/PI, and Hispanic/Latino/a that were bilingual Spanish/English speakers received a list each week of racially, ethnically, or linguistically concordant patients with upcoming or recently completed clinic visits at FHCC. Using these lists, navigators proactively contacted patients in their priority population to offer services, assess immediate needs, and provide contact information. Patients who accepted the navigation services at the initial contact received follow-up phone calls by the navigator in the first week or two; ongoing follow-up varied depending on patients’ needs.

## Methods

### Study design

We conducted an observational explanatory mixed methods study, where quantitative data were collected and analyzed first, followed by qualitative data to further inform quantitative findings (Creswell & Creswell, 2017). Patients who met eligibility criteria completed a baseline survey between April 2021 to June 2022 and a follow up survey between July 2021 to September 2022 to assess patient centered outcomes in those who received RELC PN, traditional PN and no PN. Following preliminary results of patient surveys, we conducted one-on-one interviews with patients who received the RELC model of PN to enhance validity and further explore findings through patient perspectives, from August 2022 to November 2022. Either model of PN was received based on patient request, a positive screening or clinical team referral. Additionally, RELC PN could also have been offered through proactive RELC navigator outreach. Patients who were not connected to a PN through one of these standard processes did not receive services and served as the comparator group.

### Study population and recruitment

We recruited patients from FHCC, a large cancer research center located in Seattle, WA. Patients were eligible (Table 1) to participate in the survey if they were 18 years or older, a new patient at FHCC (defined as being within 3 months of their first visit since 2018), AND preferred English or Spanish for medical care. They also had to have one of the following: Medicaid or no insurance OR a self-reported race that included AI/AN OR B/AA (the two priority populations for the longitudinal model of navigation when the survey launched). These final criteria, serving as proxies for low-income and potential experiences of racism and its sequelae, respectively, were included to concentrate the survey sample on patients most likely to benefit from navigation (Boyd et al., 2020). Participants were eligible for an interview if they had a clinic visit at FHCC within the past year AND self-identified as AI/AN, B/AA, AA/PI or used Spanish for medical care, and had at least 2 encounters with a patient navigator under the RELC model. Patients were identified using new patient lists that were regularly provided to the study team by Clinical Analytics staff. We followed STROBE guidelines for the observational analysis and COREQ guidelines for reporting qualitative interview data (Tong et al., 2007; von Elm et al., 2014).

### Positionality

The diverse research team was composed of research scholars, physicians, research staff, and graduate students. Team members identified as African American, Latina, Asian, and Non-Hispanic White. Several members of the team were bilingual English/Spanish speaking researchers and physicians. The research and its analyses were led in collaboration with this multiracial and multilingual study team which reflected the race, ethnicities, and linguistic preferences of participants included in the study. To ensure all perspectives were considered and included, team members met bi-weekly to discuss the analysis and all drafts of the manuscript.

### Patient surveys

Baseline and follow-up surveys assessed patient demographics and validated measures of patient satisfaction with care, healthcare-related discrimination, perceived stress, and resilience (Table 2) (Ezzati et al., 2014; Jean-Pierre et al., 2011; Patient Navigation Research Program Group, 2012; Remor, 2006; Vaishnavi et al., 2007; Weech-Maldonado, Dreachslin, et al., 2012; Weech-Maldonado, Hall, et al., 2012). A follow-up survey, completed 3–5 months after the first, repeated the same measures from the baseline survey and assessed attitudes and experiences related to navigation services. Our goal sample size for this pilot study was 160 baseline surveys and 120 follow-up surveys.

Participants were invited to participate via mailed letter and email (if available). The invitation included a REDCap link to the informed consent information and baseline survey to complete online. For those recruited by email, up to 3 emails were sent to participants. If there was no response after 2 weeks of the last contact effort, patients were contacted via phone call up to two times if they identified with one of the priority populations (AI/AN, B/AA, Spanish speaking). Bilingual Spanish-speaking study staff and interpreters were available for patients who were Spanish-speaking.

Depending on preference, interested participants completed online or telephone screenings, provided informed consent and were directed to a questionnaire that took approximately 15 minutes to complete. For participants who preferred Spanish, a survey was administered verbally via a bilingual staff member or using a professionally translated written version. Those who completed the baseline survey were contacted 3 months later to complete a follow-up survey via email with two reminders. If there was no response after 2 weeks, patients who identified as one of the priority populations were contacted via phone call up to 3 times. Participant demographics and date of last clinic visit were obtained as part of eligibility screening from FHCC Clinical Analytics; all other information was obtained from the survey. Survey respondents received $20 for each survey they completed. All surveys were hosted and collected on Research Electronic Data Capture (REDCap) (REDCap Consortium, 2019; Harris et al., 2009).

### Patient interviews

Interviews were conducted with patients who had received the RELC model, by author CGA, a bilingual English/Spanish speaking researcher. This was done to elucidate findings from the quantitative survey and explore patient experiences in the new model. Semi-structured interview guides were developed by the study team based on the study objectives and to further explain initial survey findings. The guide was drafted by one author (KCL), then reviewed and revised by others and pilot tested. It was iteratively updated over time, based on the experience of the interviewer and participants’ responses. Potential interviewees received an invitation via email and were mailed study information. The email included a link to complete a two-question screening questionnaire, a consent form, and options to select the best days/times for study staff to call, text, or email about scheduling a consent consultation and the interview. Each participant answered two screening questions, one confirming they had received navigation services, and another about recalling their experience with the patient navigator. Those who answered “No” to either question was deemed ineligible. If after two weeks there was no response, study staff followed up with potential participants via phone call and invited them to complete eligibility screening and participate. Reasons potential interviewees did not participate included, but were not exclusive to; declination, no response after multiple contacts, passing away during the recruitment window and barriers around interpreter use. Twenty-nine semi-structured interviews were conducted over the phone in English and Spanish by bilingual study staff. Interviews lasted approximately 15–20 minutes and patients received $50 in cash for their participation. The one-on-one interviews were audio recorded, professionally transcribed, and translated (Spanish only) and de-identified prior to analysis. Data saturation was determined after the process of data collection and during thematic analysis.

## Analysis

### Patient surveys

We used descriptive statistics to characterize survey participants and non-participants by demographic, social, and clinical characteristics. Among survey participants, sociodemographic characteristics were described by navigation exposure, described as having had an appointment in person or over the phone with a navigator, using measures of frequency. We determined the prevalence of participants that reported discrimination and discussed clinical trials with a provider at baseline and follow-up. Changes in mean and standard deviation were calculated for patient satisfaction, healthcare discrimination, resilience, perceived stress, and trust. Given the small sample of participants that received the RELC model of PN (*n* = 5) who completed both baseline and follow-up surveys, we were unable to perform formal statistical tests of proportions or means and did not adjust our estimates for clinical or demographic factors.

### Patient interviews

Audio recordings of interviews were professionally transcribed verbatim. Thematic analysis procedures were used to identify concepts and key ideas about patient experiences (Creswell & Clark, 2017; Creswell & Creswell, 2017). The analysis team, consisting of three study members (EC, AT, CGA), memoed fifteen transcripts. Each memo was reduced to a central phrase, grouped into categories, and linked to a common idea. Categorized and reduced memos became preliminary codes. A codebook was created that included code names, code definitions, and an exemplar quote for each code. To establish subjective intercoder agreement, all three analysts coded the same transcript and discussed code application agreement and disagreements. Three rounds of subjective intercoder agreement, each using a different transcript, were conducted and any discrepancies were discussed and resolved. The codebook was further refined based on discussions, and code definitions updated.

After consensus had been reached, the remaining twenty-six transcripts were then divided by the analysis team to code independently (Guest et al., 2011). All transcripts were coded using Dedoose Software (Goel et al., 2022)Zubizarreta et al., 2025. During and after the coding process, the analysis team including the lead researcher (VH), reviewed the codebook to further refine codes and clarify definitions. After coding was completed, quotes for each code were exported and summarized to produce narrative code summaries. These summaries were then shared with the study team. The code summaries were then reviewed to develop potential themes and recommendations based on the participants’ experiences which were then used to explain survey results (Felner & Henderson, 2022). We then compared survey and interview findings to assess whether qualitative data converged, expanded, or diverged from quantitative findings (Creswell & Creswell, 2017). The coding process confirmed that existing categories were well-developed, clearly defined, and supported by rich participant narratives. Participants did not provide feedback on findings.

## Results

### Survey results

One-hundred eighteen people completed both the baseline and follow-up surveys out of 1,189 eligible individuals identified from FHCC clinic lists (overall response 10%). Compared to non-respondents, participants were more likely to be non-Hispanic White (57% v. 48%) (Table 3). We did not observe differences between respondents and non-respondents in terms of median age, insurance, or preferred language. Of the 118 survey respondents, 5 received the new, RELC model of navigation, 17 received the existing, traditional acute model, and 96 did not receive navigation (Table 3).

**Figure 1.**
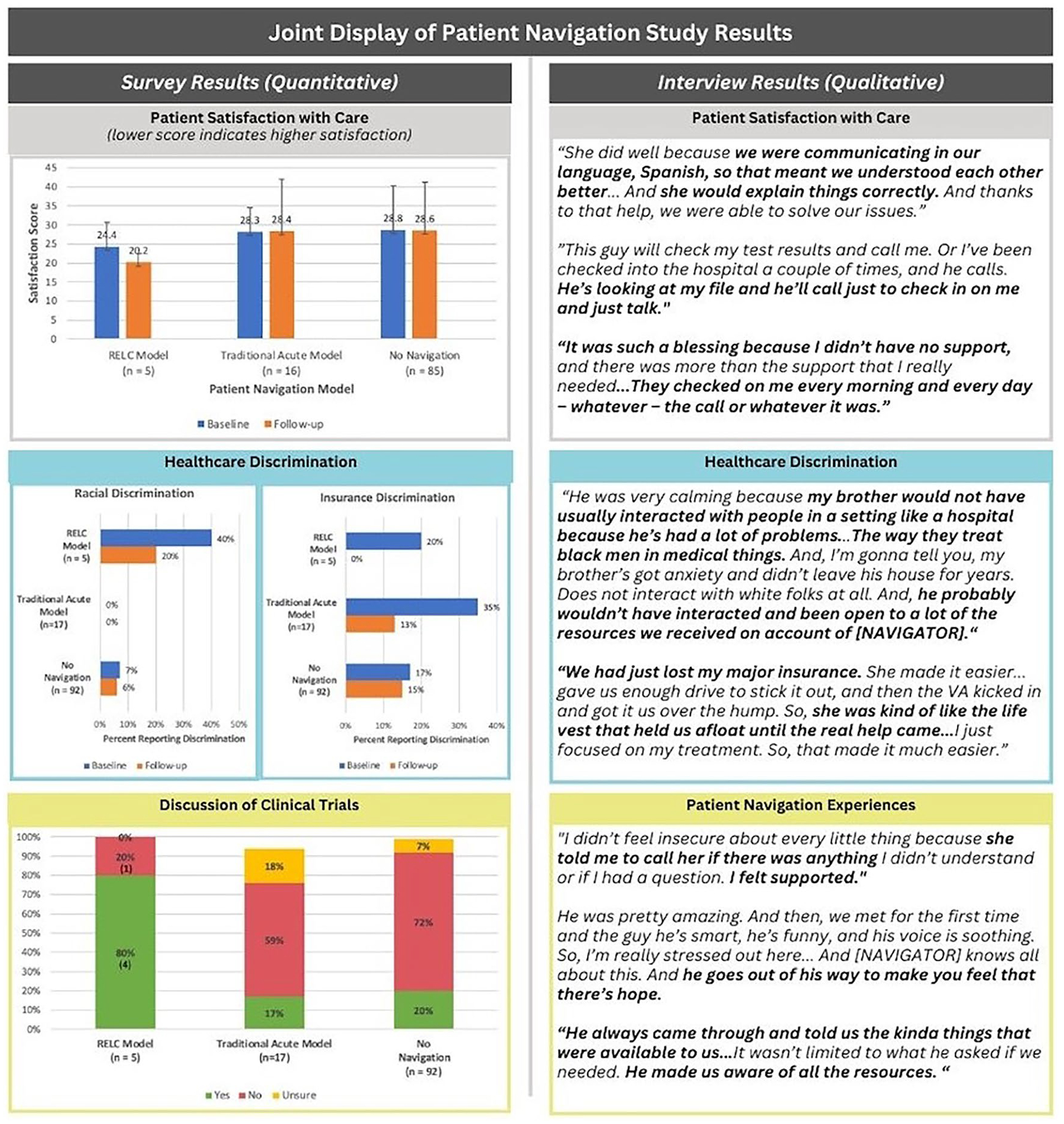
Joint Display of Patient Navigation Study Results.

Satisfaction with care improved by 4.2-points (SD 7.0) on an 18-item 5-point Likert scale (“1 = Strongly Agree” to “5 = Strongly Disagree”) among participants that received RELC navigation. No considerable change was observed for participants in the traditional or no navigation groups (Table 4). Healthcare discrimination based on race was reported by 40% (*n* = 2) of participants at baseline and 20% (*n* = 1) at follow-up of those in the RELC model (Figure 1). Additionally, 7% (*n* = 7) and 6% (*n* = 6) of participants in the non-navigated group reported racial discrimination at baseline and follow-up, but no participants in the traditional navigation group reported racial discrimination at either time point. In follow-up surveys, no participants in the RELC model reported discrimination based on insurance, while 13% (*n* = 2) of patients receiving the traditional model, and 15% (*n* = 14) of patients receiving no navigation reported insurance discrimination at follow-up.

Eighty percent (*n* = 4) of participants that received RELC navigation reported discussing clinical trials with a provider, compared to 17% (*n* = 3) that received the traditional acute, episodic navigation and 20% (*n* = 19) of participants that were not navigated (Figure 1). No differences over time or by exposure group were observed for resilience or perceived stress (data not shown).

### Interview results

We conducted a total of twenty-nine interviews with patients who received the new RELC model of navigation. Participants identified as being B/AA (38%; *n* = 11), Hispanic or Latino (38%; *n* = 11), AI/AN (17%; *n* = 5), and AA/PI (7%; *n* = 2). More than half of participants spoke English (62%; *n* = 18) and the rest spoke Spanish (38%; *n* = 11) (Table 5). Eight themes were identified that were related to three focus areas (Table 6).

1. *Communication and relationships*. Patients discussed how language and racial concordance between patients and navigators facilitated barrier resolution, communication, and increased patient comfort and trust. Communication with navigators was often described as effective, consistent, and respectful, which facilitated positive and trusting relationships between navigators and patients.
2. *Addressing barriers and resource/service provision*. The assistance provided by navigators to patients was extensive and included psychosocial support, linkages to resources for financial support, food and housing insecurity, transportation, appointment scheduling, medication payment programs, language barriers, and assisting caregivers. Patient navigators were instrumental in helping patients with these barriers, providing much needed assistance, emotional support, advocacy, and care coordination. This reduced patient stress, allowed patients to focus on their treatment, increased their skills in navigating the healthcare and social service systems, and increased their comfort in the healthcare setting.
3. *Patients’ perceptions and experience*. Repeatedly, patients shared positive feedback about the navigation services provided and a desire for them to continue. Many praised navigators’ efficiency, kindness, responsiveness, helpfulness, respectful communication, and professionalism. However, a few participants expressed concerns, including challenges reaching their navigator, lack of follow up, and misalignment of provided resources with needs.

## Mixed methods results

### Patient satisfaction

Analysis of patient-reported satisfaction with care demonstrated an improvement among participants that received RELC model navigation, while no observable change was noted for participants in the traditional acute or no navigation groups (Figure 1). Interviews with patients in the RELC navigation model demonstrated that patients overwhelmingly had positive experiences with the navigation services provided, citing navigators’ efficiency, helpfulness, and professionalism. Participants described their navigator as going above and beyond to make them feel that there was hope, helping them feel understood, and helping them feel more confident navigating systems and resources. A common theme that emerged was that patient navigators helped patients to access needed resources and made them feel more at ease and supported in their medical care.

### Racial and insurance discrimination

Analysis of CAHPS Healthcare Discrimination survey responses revealed a potential reduction in racial and insurance discrimination in subsequent follow-up surveys among patients receiving the RELC navigation model (Figure 1). Similar but smaller reductions in the proportion of reporting insurance discrimination was also observed among patients receiving the traditional acute model of navigation and no navigation. While these quantitative findings are informative, the small sample size limits our ability to draw definitive conclusions. However, findings from patient interviews seemed to support this finding: participants in the RELC navigation model emphasized that racism was a significant barrier to cancer treatment and discussed that patient navigators were instrumental in helping them to overcome these barriers during treatment. The RELC longitudinal model also matched patients to navigators from similar racial and ethnic backgrounds and language, and appreciation for concordance with navigators was a recurring theme in interviews. Several patients reported that concordance improved comfort, trust, and communication.

### Clinical trials

Our survey results demonstrated that most of the patients who received the RELC navigation model recalled discussing clinical trials with their provider, whereas about a fifth of patients in the traditional acute and no navigation models reported discussing clinical trials (Figure 1). While discussing clinical trials was not explicitly part of the navigators’ role, these discussions may have been supported by the work navigators did to foster trust and support clinical communication. For example, an important theme that arose in patient interviews with those that had received RELC navigation was that navigators had consistently followed up with patients, providing ongoing communication, emotional support, and helped them coordinate aspects of care which fostered a sense of trust. A common sentiment echoed by several patients was that navigators gave them hope, and confidence in navigating all aspects of their care, while nurturing a sense of empowerment. Taken together, these findings may suggest the significant impact RELC navigation models may have on enhancing patient trust and communication and may be a valuable strategy in promoting participation in clinical trials among racially, ethnically, and linguistically diverse patients.

## Discussion

Our quantitative findings suggest that proactive, longitudinal, racially, ethnically and linguistically concordant PN models may be associated with higher patient satisfaction, less discrimination based on insurance, and higher likelihood of clinical trial discussions. Interviews expanded upon these findings, with patients highlighting their communication with navigators, which helped to facilitate a substantial level of comfort and trust. Participants emphasized the navigator’s kindness, efficiency, and professionalism. Some also described how they assisted with overcoming emotional, financial, and care coordination barriers, which reduced stress and allowed them to focus on their treatment. Additionally, survey findings demonstrated an increase in patient satisfaction in the RELC navigated patients at follow-up, although satisfaction was relatively low overall. Lower overall satisfaction was also discovered in a study with a control and PN group where patients had a low socioeconomic status and were from an ethnic or racial minority group (Wells et al., 2018). Greater patient satisfaction with PN is consistent with other studies that demonstrate that patient navigators can play a crucial role in providing comprehensive support and addressing patient needs (Jean-Pierre et al., 2012), including more timely care, decreased treatment costs, and better adherence and satisfaction with cancer care (Bernardo et al., 2019; Chan et al., 2023; Denver Patient Navigation Research Program, 2012; Jean-Pierre et al., 2011; Nelson et al., 2020; Post et al., 2015; Schwaderer & Itano, 2007; Wells et al., 2018; Writing Group of the Patient Navigation Research Program, 2014).

Patient interviews also revealed numerous barriers to care that navigators helped them overcome. In interviews, patients highlighted racism and language challenges as barriers that patient navigators in the RELC model helped to mitigate. These qualitative findings were consistent with survey findings which found a potential decrease in racial and insurance discrimination at follow-up for participants in this new model. This suggests a possible mechanism by which linguistic and racial concordance between patients and navigators can positively impact care through reduced bias. Several others have shown that concordance can impact discrimination within the cancer care setting, which can contribute to improved care timeliness, patient trust, satisfaction, and patient outcomes (Blanchard et al., 2007; Charlot et al., 2015; Loeb et al., 2023; Lor & Martinez, 2020; Schenker et al., 2010; Seible et al., 2021; Traylor et al., 2010). Future work should investigate the extent to which changes in experienced discrimination may mediate the relationship between linguistic and racial concordance and improved outcomes.

In our study, language and racial concordance with navigators improved patient trust and facilitated positive communication, which empowered patients to feel confident in navigating the healthcare setting. We speculate that this supportive environment may have helped to facilitate discussions of clinical trial options with patients and may have contributed to our finding that navigated patients in either the RELC or the traditional acute model more often had discussions about clinical trials than non-navigated patients. Several studies have demonstrated that PN lowers rates of clinical trial refusal and improves patient knowledge and understanding of the clinical study and their participation, especially among minoritized patients (Cartmell et al., 2016; Fouad et al., 2016; Ghebre et al., 2014; Holmes et al., 2012). This demonstrates the potential of PN and of concordant navigation models to improve diversity in clinical trial participation which can address issues of equity and inclusivity in research.

### Limitations

This study has several limitations. First, a smaller-than-expected number of navigated patients completed baseline and follow-up surveys. As a result, we were unable to conduct reliable statistical comparisons between groups. This was possibly due to the study recruitment being conducted during the COVID-19 pandemic. The small sample provides some preliminary insight into patient experience with different navigation models but significantly limits our ability to draw definitive conclusions. As a result, an explanatory sequential mixed methods study was conducted to improve validity and better understand patient experiences with the new RELC model. Furthermore, because navigation type was not randomly assigned, there may be unmeasured differences between groups, such as factors influencing whether a patient was offered, sought out, or accepted navigation support that could contribute to the observed differences in survey responses. Second, study inclusion was restricted to those that either spoke English or Spanish due to institutional policies that require a witness for consent by a non-English speaker. Participants were required to find a bilingual individual to witness their consent in addition to the interpreter, which systematically excluded those without close bilingual contacts who were available during the consent process. This limits the generalizability of our findings as Spanish was the only non-English language represented. Future studies should aim to include more language groups to expand representation and improve insight into the perspectives of other linguistic communities. Studies would benefit greatly from language diversity which can further represent these populations.

## Conclusion

Our study highlights the potential of a proactive, RELC PN model to improve patient satisfaction and participation in clinical trials and reduce patient experiences of racism and insurance discrimination. Interviews with patients highlight the barriers faced by racially and ethnically minoritized populations during cancer treatment and the importance of concordant PN support during treatment. While our survey data provided some preliminary insight into the potential of this RELC model, a larger study is needed to confirm these findings. A larger sample size and randomized assignment of navigation models would also allow for statistical comparisons and strengthen causal interpretation. Further research is also needed to explore what may make this model effective, examine strategies for how it may be implemented effectively, and understand how concordance-based approaches might contribute to improved patient outcomes, while advancing equity and patient satisfaction and care.

## Figures and Tables

**Table 1.** Eligibility: inclusion criteria for survey and interview participants.

Eligibility survey	Eligibility interview
New patient ≥ 18 years at FHCC	Patient ≥ 18 years who had a clinic visit at FHCC within the past year
**AND**	**AND**
Prefer English or Spanish for medical care	Had 2+ encounters with patient navigator under new culturally, racially and linguistically concordant model
**AND** at least 1 of the following:	**AND** at least 1 of the following:
Medicaid insurance or no insurance Self-reported race that includes American Indian/Alaskan Native or Black/African American	Use Spanish for medical care Self-reported race that includes American Indian/Alaskan Native, Black/African American or Asian American/Pacific Islander

**Table 2.** Survey measures: items analyzed in patient questionnaire.

Construct	Measure and references	Description
Satisfaction with cancer care	*Patient Satisfaction with Cancer-Related Care* (Barocas et al., 2014; White-Means & Osmani, 2017)	18-item measure of satisfaction with cancer care, including aspects of communication with providers, confidence with self-management, and care coordination; responses on a 5-point Likert scale
Healthcare discrimination	*CAHPS Healthcare Discrimination* (Creswell & Creswell, 2017; Tong et al., 2007)	2-item measure with a 4-point Likert scale, gauging frequency of experiencing healthcare discrimination related to insurance type or race and ethnicity
Stress	*Perceived Stress Scale—Short Form* (Benjamins et al., 2016; Yedjou et al., 2017)	4-item measure of perceived degree of life-stress, with response options of never, almost never, sometimes, fairly often, and very often
Resilience	*Connor-Davidson Resilience Scale 2 (CD-RISC2)* (Boyd et al., 2020)	2 items measuring resilience, on a 5-point Likert scale
COVID-19	*WHO COVID-19 questions*	11 questions regarding exposure to COVID-19, behavioral response to the pandemic, and work-related impact

**Table 3.** Demographics: description of survey respondents and non-respondents.

	Participants (*n* = 118)		Email only recruited non-participants (*n* = 723)		Email and telephone recruited non-participants (*n* = 281)		All non-participants (*n* = 1004)	
Characteristic	*n*	%	*n*	%	*n*	%	*n*	%
Age (median, IQR)	51	25	52	23	54	22	52	23
Race and ethnicity								
NH White	67	57	457	66	9	3.2	484	48
NH Black	15	13	17	2	149	53	166	17
NH AI/AN	10	8	5	1	51	18	56	6
NH AA/PI	3	3	65	9	0	0	65	6
Hispanic or Latino (any race)	14	12	53	7	56	20	109	11
Both unknown	7	6	60	8	2	1	62	6
Race or ethnicity unknown	2	2	48	7	14	5	62	6
Insurance								
Commercial	13	11	10	1	97	35	107	11
Medicaid	54	46	369	51	80	28	449	45
Medicare	6	5	3	<1	51	18	54	5
Tricare	0	0	2	<1	3	1	5	<1
Other	3	3	1	<1	9	3	10	1
Self-Pay	42	36	338	47	41	15	379	38
Language								
English	114	97	721	99	240	85	961	96
Spanish	4	3	2	<1	41	15	43	4

**Table 4.** Satisfaction with care: comparison in patient satisfaction among RELC, traditional navigation, and no navigation groups at baseline and follow-up.

Navigation model	RELC model of patient navigation (*n* = 5)		Traditional acute patient navigation (*n* = 16)		No patient navigation (*n* = 85)	
	*μ*	sd	*μ*	sd	*μ*	sd
Baseline	24.4	6.2	28.3	6.3	28.8	11.4
Follow-up	20.2	2.2	28.4	13.6	28.6	12.7
Δ	−4.2	7.0	0.2	13.7	−0.2	10.9

**Table 5.** Interview participant demographics.

Demographics	Participants (*n* = 29)	
	*n*	%
Race and ethnicity		
Non-Hispanic Black/African American	11	38
American Indian/Alaska Native	5	17
Asian and Pacific Islander	2	7
Hispanic or Latino/a	11	38
Language		
English	18	62
Spanish	11	38

**Table 6.** Common ideas identified across interviews subdivided into focus areas, themes and supportive quotes.

Themes	Representative quotes
Focus Area 1: Communication and relationships	
**Theme 1.** Language and racial concordance between patients and navigators facilitate barrier resolution, communication, and increases patient comfort and trust.	*“*…*we were communicating in our language, Spanish, so*…*we understood each other better*…*she would talk to the lady in English, and then [the navigator] would relay that information to us*…*thanks to that help, we were able to solve our issues*…*”*
**Theme 2.** Effective, consistent, and respectful communication facilitated positive and trusting relationships between navigators and patients. However, communication with navigators sometimes varied.	*“[The navigator] was accountable to us. Making phone calls and looking something up*… *Talking to a doctor*… *[and] navigator. He made sure that he knew what he was committing to*…*we’d have a time to check in*… *we would just be accountable for the information and to each other. Like, I did my part and he did his part*…*”*
**Theme 3.** Navigator roles, responsibilities, and available resources were sometimes not consistently communicated to patients throughout their navigation services which resulted in some confusion among patients.	*“*…*it’s not [the navigator’s] job because I do have a social worker, but if*…*they could help with housing vouchers or finding low-income housing for people*…*that would help a lot. I don’t think they provide that service, but*…*that didn’t mean that [the navigator] didn’t try.”*
Focus Area 2: Addressing barriers and resource/service provision	
**Theme 4.** Navigator assistance to patients is extensive and not only includes psychosocial support, but also financial support, food, housing, transportation, appointment scheduling, medication payment, language assistance, and assisting caregivers.	*“I was having financial troubles*…*So, he was able to get me some gift cards from Safeway, which I didn’t have money for food*…*And that helped so much*…*I didn’t have to worry about food*…*And last year he got me, I was getting it monthly, and he got me a couple of cards*…*And it just really helped.”*
**Theme 5.** Financial barriers, distance to treatment, and racial discrimination were cited as significant barriers to cancer treatment. Patient navigators were instrumental in helping patients with these barriers.	*“*…*my brother would not have usually interacted with people in*…*a hospital because he’s had a lot of*…*disparities. The way they treat Black men in medical things*…*[the navigator] gave him a sense of peace*…*and eased his heart and gave him reassurance*…*that if [he] wasn’t comfortable with something yet, he would be his voice. So, that made my brother comfortable right away*…*my brother’s got anxiety and didn’t leave his house for years. Does not interact with white folks at all. And he probably wouldn’t have interacted and been open to a lot of the resources we received [from the navigator].”*
**Theme 6.** Navigator services reduced patient stress. Navigators provided linkages to resources, emotional support, advocacy, and care coordination, which allowed patients to focus on their treatment.	*“When I told her I was doing this procedure or I’m busy*…*she would just say, “When’s a good time to call?” and then we would adjust*…*she actually talked to my wife half the time because I wasn’t in the right state of mind. And both of those things just made it easier for me. Because, really, it just came down so I could focus on getting better.”*
Focus Area 3: Patients’ perceptions and experience	
**Theme 7.** Overall, patients had positive experiences with the navigation services provided. Many expressed a desire for navigation services to continue. Many praised navigators’ kindness, responsiveness, and professionalism.	*“*.…*he’s smart, he’s funny, and his voice is soothing. So, I’m really stressed out here. I’m dealing with stage four bone cancer, and they found three more lesions on my spine. And [the navigator] knows all about this. And he goes out of his way to make you feel that there’s hope. How did you know I was going to get choked up?”*
**Theme 8.** A few participants expressed concern with the navigation services they received. Concerns include challenges reaching their navigator, lack of follow up on continued needs, and misalignment of resources with their expressed needs.	*“And I talked to [the navigator]. He said, “We’ll get that information.”* …*And they said, “*…*They have places that we can help you get into.” And that fell through. So, I was really hurt, and upset, and disappointed*…*It started off as a good experience and I trusted what they were saying. And that didn’t turn out very well for me*… *then I just stopped hearing from them.”*
